# Supplementary material for: Prevalence of Acute Hepatitis E Virus Infections in Swiss Blood Donors 2018–2020
Source: Viruses. 2024 May 8;16(5):744. doi: 10.3390/v16050744 (PMC11125967; doi:10.3390/v16050744)

**Table S1:** Overview of the HEV sequences, case numbers and deposited accession numbers used for phylogenetic analysis. HEV genotype as described in HEVNet genotyping tool: https://www.rivm.nl/mpf/typingtool/hev/. Case Number nomenclature: e.g., DOxxCAYYMM; (donor/number/canton/year/month)

| Number | Case Number | Accession Nı | HEV subgenotype / cla | Name, Smith DB |
|---|---|---|---|---|
| 1 | D013BE1810 | OV844702 | 3c / clade 3abchijklm | 3c |
| 2 | D014BE1810 | OV844703 | 3h3 / clade 3abchijklm | 3h |
| 3 | D015D1810 | OV844704 | 3c / clade 3abchijklm | 3c |
| 4 | D016BS1810 | OV844705 | 3_uc3 / clade 3abchijklm | 3 unclassified |
| 5 | D017BE1811 | OV844706 | 3c / clade 3abchijklm | 3c |
| 6 | D018TG1810 | OV844781 | 3h3 / clade 3abchijklm | 3h |
| 7 | D019BE1811 | OV844707 | 3e / clade 3efg | 3e |
| 8 | D021BE1811 | OV844708 | 3c / clade 3abchijklm | 3c |
| 9 | D022SG1811 | OV844709 | 3c / clade 3abchijklm | 3c |
| 10 | D024AR1811 | OV844710 | 3c / clade 3abchijklm | 3c |
| 11 | D025D1811 | OV844711 | 3c / clade 3abchijklm | 3c |
| 12 | D026SG1811 | OV844787 | 3h3 / clade 3abchijklm | 3h |
| 13 | D027ZH1812 | OV844798 | 3h3 / clade 3abchijklm | 3h |
| 14 | D028FR1812 | OV844772 | 3f1 / clade 3efg | 3f |
| 15 | D029VS1812 | OV844712 | 3h3 / clade 3abchijklm | 3h |
| 16 | D030SG1812 | OV844713 | 3h3 / clade 3abchijklm | 3h |
| 17 | D031AG1812 | OV844762 | 3c / clade 3abchijklm | 3c |
| 18 | D032VS1812 | OV844714 | 3c / clade 3abchijklm | 3c |
| 19 | D033VS1901 | OV844715 | 3h3 / clade 3abchijklm | 3h |
| 20 | D034GE1901 | OV844716 | 3h3 / clade 3abchijklm | 3h |
| 21 | D035D1901 | OV844717 | 3c / clade 3abchijklm | 3c |
| 22 | D036OW1901 | OV844774 | 3 / clade 3abchijklm | 3 |
| 23 | D037BS1901 | OV844718 | 3h3 / clade 3abchijklm | 3h |
| 24 | D038SO1901 | OV844766 | 3f1 / clade 3efg | 3f |
| 25 | D039AG1901 | OV844767 | 3h3 / clade 3abchijklm | 3h |
| 26 | D041BE1902 | OV844719 | 3c / clade 3abchijklm | 3c |
| 27 | D042ZH1902 | OV844803 | 3h3 / clade 3abchijklm | 3h |
| 28 | D043BE1902 | OV844720 | 3c / clade 3abchijklm | 3c |
| 29 | D044AG1902 | OV844768 | 3h3 / clade 3abchijklm | 3h |
| 30 | D045F1902 | OV844721 | 3f1 / clade 3efg | 3f |
| 31 | D046VS1902 | OV844722 | 3c / clade 3abchijklm | 3c |
| 32 | D047BL1902 | OV844723 | 3h3 / clade 3abchijklm | 3h |
| 33 | D048ZH1902 | OV844804 | 3h3 / clade 3abchijklm | 3h |
| 34 | D050ZH1902 | OV844805 | 3h3 / clade 3abchijklm | 3h |
| 35 | D051TG1902 | OV844782 | 3ra | 3ra |
| 36 | D052NE1902 | OV844778 | 3h3 / clade 3abchijklm | 3h |
| 37 | D053VS1904 | OV844724 | 3 / clade 3abchijklm | 3 |
| 38 | D054AG1904 | OV844769 | 3c / clade 3abchijklm | 3c |
| 39 | D056FR1904 | OV844725 | 3h3 / clade 3abchijklm | 3h |
| 40 | D057AG1904 | OV844770 | 3c / clade 3abchijklm | 3c |
| 41 | D058BL1904 | OV844726 | 3c / clade 3abchijklm | 3c |
| 42 | D060SG1905 | OV844727 | 3c / clade 3abchijklm | 3c |
| 43 | D061AG1905 | OV844771 | 3h3 / clade 3abchijklm | 3h |

| | | | | |
|---|---|---|---|---|
| 44 | D063ZH1905 | OV844806 | 3c / clade 3abchijklm | 3c |
| 45 | D065BE1905 | OV844728 | 3c / clade 3abchijklm | 3c |
| 46 | D066BL1905 | OV844729 | 3h3 / clade 3abchijklm | 3h |
| 47 | D067F1906 | OV844730 | 3c / clade 3abchijklm | 3c |
| 48 | D068BE1906 | OV844731 | 3c / clade 3abchijklm | 3c |
| 49 | D069BS1906 | OV844732 | 3l clade 3abchijklm | 3l |
| 50 | D070VD1906 | OV844733 | 3h3 / clade 3abchijklm | 3h |
| 51 | D072VD1906 | OV844734 | 3h3 / clade 3abchijklm | 3h |
| 52 | D073BE1907 | OV844735 | 3c / clade 3abchijklm | 3c |
| 53 | D077BE1907 | OV844736 | 3c / clade 3abchijklm | 3c |
| 54 | D079GE1907 | OV844737 | 3c / clade 3abchijklm | 3c |
| 55 | D080ZH1907 | OV844783 | 3h3 / clade 3abchijklm | 3h |
| 56 | D081VD1908 | OV844738 | 3c / clade 3abchijklm | 3c |
| 57 | D082VD1908 | OV844739 | 3ra | 3ra |
| 58 | D083VS1908 | OV844740 | 3f1 / clade 3efg | 3f |
| 59 | D084TG1908 | OV844807 | 3c / clade 3abchijklm | 3c |
| 60 | D085AG1908 | OV844763 | 3h3 / clade 3abchijklm | 3h |
| 61 | D087NE1909 | OV844779 | 3h3 / clade 3abchijklm | 3h |
| 62 | D086ZH1909 | OV844788 | 3c / clade 3abchijklm | 3c |
| 63 | D088ZH1909 | OV844789 | 3c / clade 3abchijklm | 3c |
| 64 | D089GE1909 | OV844741 | 3f1 / clade 3efg | 3f |
| 65 | D090LU1909 | OV844775 | 3c / clade 3abchijklm | 3c |
| 66 | D091ZH1909 | OV844790 | 3c / clade 3abchijklm | 3c |
| 67 | D092D1910 | OV844791 | 3c / clade 3abchijklm | 3c |
| 68 | D093GE1910 | OV844742 | 3c / clade 3abchijklm | 3c |
| 69 | D095SG1911 | OV844792 | 3h3 / clade 3abchijklm | 3h |
| 70 | D096ZH1911 | OV844793 | 3f1 / clade 3efg | 3f |
| 71 | D098TI1911 | OV844784 | 3ra | 3ra |
| 72 | D099F1912 | OV844743 | 3f1 / clade 3efg | 3f |
| 73 | D101VD1912 | OV844744 | 3h3 / clade 3abchijklm | 3h |
| 74 | D102SG1912 | OV844745 | 3h3 / clade 3abchijklm | 3h |
| 75 | D103SG1912 | OV844746 | 3h3 / clade 3abchijklm | 3h |
| 76 | D104SO1912 | OV844764 | 3h3 / clade 3abchijklm | 3h |
| 77 | D105LU1912 | OV844747 | 3h3 / clade 3abchijklm | 3h |
| 78 | D107ZG1912 | OV844776 | 3f1 / clade 3efg | 3f |
| 79 | D108AG1912 | OV844794 | 3h3 / clade 3abchijklm | 3h |
| 80 | D109VS2001 | OV844748 | 3h3 / clade 3abchijklm | 3h |
| 81 | D110ZG2001 | OV844777 | 3l clade 3abchijklm | 3l |
| 82 | D111TI2001 | OV844785 | 3h3 / clade 3abchijklm | 3h |
| 83 | D112GR2001 | OV844773 | 3h3 / clade 3abchijklm | 3h |
| 84 | D113BL2001 | OV844749 | 3h3 / clade 3abchijklm | 3h |
| 85 | D114BS2001 | OV844750 | 3h3 / clade 3abchijklm | 3h |
| 86 | D115AG2001 | OV844765 | 3ra | 3ra |
| 87 | D117BE2002 | OV844751 | 3h3 / clade 3abchijklm | 3h |
| 88 | D118VD2002 | OV844752 | 3f1 / clade 3efg | 3f |
| 89 | D119JU2002 | OV844780 | 3h3 / clade 3abchijklm | 3h |
| 90 | D120SZ2002 | OV844795 | 3 / clade 3abchijklm | |
| 91 | D121ZH2002 | OV844796 | 3h3 / clade 3abchijklm | 3h |

| | | | | |
|---|---|---|---|---|
| 92 | D122GE2002 | OV844753 | 3c / clade 3abchijklm | 3c |
| 93 | D123ZH2002 | OV844797 | 3h3 / clade 3abchijklm | 3h |
| 94 | D124BL2003 | OV844754 | 3c / clade 3abchijklm | 3c |
| 95 | D125LU2004 | OV844755 | 3h3 / clade 3abchijklm | 3h |
| 96 | D126TI2004 | OV844786 | 3c / clade 3abchijklm | 3c |
| 97 | D128GE2005 | OV844756 | 3e / clade 3efg | 3e |
| 98 | D129VD2005 | OV844757 | 3c / clade 3abchijklm | 3c |
| 99 | D130SG2004 | OV844799 | 3h3 / clade 3abchijklm | 3h |
| 100 | D131ZH2005 | OV844800 | 3c / clade 3abchijklm | 3c |
| 101 | D132SG2005 | OV844758 | 3c / clade 3abchijklm | 3c |
| 102 | D133BE2006 | OV844759 | 3a / clade 3abchijklm | 3a |
| 103 | D134LU2006 | OV844801 | 3f1 / clade 3efg | 3f |
| 104 | D135BE2006 | OV844760 | 3ra | 3ra |
| 105 | D138ZH2008 | OV844802 | 3c / clade 3abchijklm | 3c |
| 106 | D140SG2009 | OV844761 | 3c / clade 3abchijklm | 3c |

**Table S2:** Comparison of the HEV RNA Assays, minipool sizes and HEV RNA prevalence stratified by testing centre.

| Testing centre | HEV RNA Assay | Minipool size | Number donations | Number HEV RNA positive | HEV RNA prevalence |
|---|---|---|---|---|---|
| Aarau | Grifols Procleix | 15 | 42,761 | 10 | 1:4,276 |
| Lugano | Grifols Procleix | 12 | 20,082 | 4 | 1:5,020 |
| Berne | Roche cobas | 16 | 287,450 | 74 | 1:3,884 |
| La-Chaux-de-Fonds | Roche cobas | 24 | 35,302 | 4 | 1:8,825 |
| Zurich | Roche cobas | 24 | 152,141 | 33 | 1:4,610 |

**Table S3:** The HEV RNA concentration stratified by HEV subgenotype detected.

| | HEV 3c clade 3abchij | HEV 3h3 clade 3abchij | HEV 3f1 clade 3efg | HEV 3ra |
|---|---|---|---|---|
| Mean VL | 110,596 IU/ml | 71,789 IU/ml | 69,883 IU/ml | 968 IU/ml |
| Median VL | 1,700 IU/ml | 947 IU/ml | 4,118 IU/ml | 230 IU/ml |
| Geometric mean VL | 1,692 IU/ml | 1,223 IU/ml | 3,095 IU/ml | 334 IU/ml |
| Number cases | 40 | 42 | 10 | 5 |

The only mean VL between the genotypes which appears significantly different appears to be the HEV 3ra. As we do not know the exact primer binding sites of the screening and confirmatory HEV RNA assays it is impossible to determine if this rabbit genotype has generally lower viral loads or if this is a technical problem of the assays.

**Figure S1:** Linear phylogenetic analysis of the HEV variants from Swiss blood donors, October 2018 - September 2020 with bootstrap values.

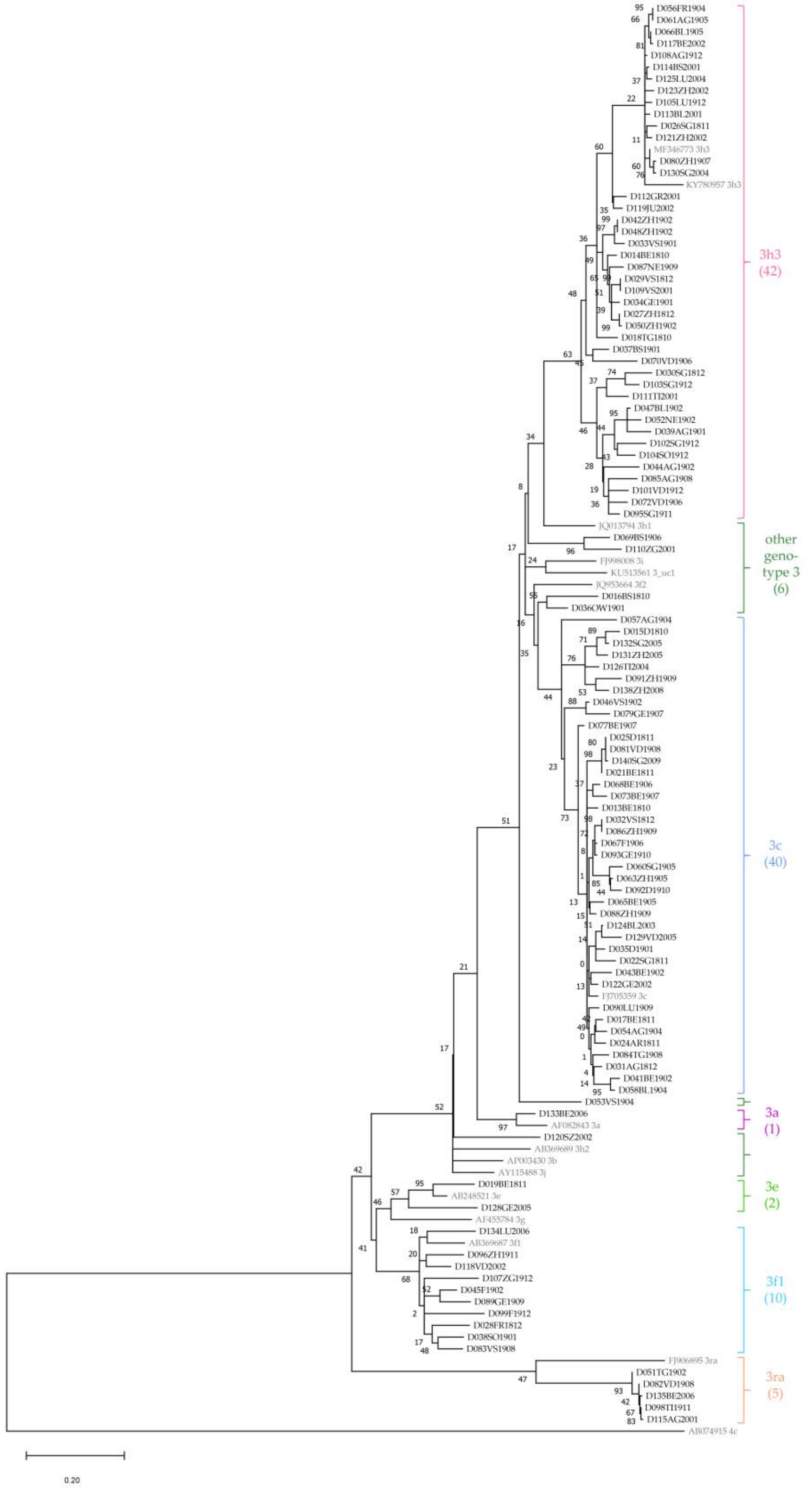

Supplement: Supplementary file 1 [file viruses-16-00744-s001.zip › viruses-2940673-supplementary.pdf]
